# CAR T-cells for colorectal cancer immunotherapy: Ready to go?

**DOI:** 10.3389/fimmu.2022.978195

**Published:** 2022-11-15

**Authors:** Bouchra Ghazi, Adil El Ghanmi, Sarah Kandoussi, Amina Ghouzlani, Abdallah Badou

**Affiliations:** ^1^ Faculty of Medicine, Mohammed VI University of Health Sciences (UM6SS), Casablanca, Morocco; ^2^ Mohammed VI International University Hospital, Faculty of Medicine, Mohammed VI University of Health Sciences (UM6SS), Casablanca, Morocco; ^3^ Immuno-Genetics and Human Pathology Laboratory, Faculty of Medicine and Pharmacy, Hassan II University, Casablanca, Morocco

**Keywords:** colorectal cancer, CAR T cells, immunotherapies, tumor microenvironement, tumor mutational burden, clinical data

## Abstract

Chimeric antigen receptor (CAR) T-cells represent a new genetically engineered cell-based immunotherapy tool against cancer. The use of CAR T-cells has revolutionized the therapeutic approach for hematological malignancies. Unfortunately, there is a long way to go before this treatment can be developed for solid tumors, including colorectal cancer. CAR T-cell therapy for colorectal cancer is still in its early stages, and clinical data are scarce. Major limitations of this therapy include high toxicity, relapses, and an impermeable tumor microenvironment for CAR T-cell therapy in colorectal cancer. In this review, we summarize current knowledge, highlight challenges, and discuss perspectives regarding CAR T-cell therapy in colorectal cancer.

## Introduction

State-of-the-art in cancer immunotherapy consists of approaches that combine cellular engineering with synthetic biology to generate a powerful immune weapon. Current strategies include therapeutic tumor-associated antigen (TAA)-specific monoclonal antibodies, immune checkpoint inhibitors (ICIs) (1–4), bispecific antibodies, and four generations of chimeric antigen receptor (CAR) T-cells for targeting multiple tumor antigens. All these strategies are widely used in clinical practice for treating hematologic and solid tumors.

Colorectal cancer (CRC) currently ranks third in cancer incidence and continues to be the leading cause of cancer-related death. In 2020, there were 1,931,590 new cases of CRC worldwide and 935,173 deaths, accounting for 10.0% and 9.4% of the total number of cancer cases and deaths, respectively (5, 6). In CRC, the most effective therapeutic strategy for increasing patient survival involves surgical resection alone or in combination with chemotherapy and radiotherapy. However, in advanced stages of CRC, representing approximately 25% of cases, therapeutic strategies based on surgery are not useful and are associated with higher risk of poor outcomes (7, 8).

Current pharmacological approaches for optimizing CRC management include non-specific drugs, such as fluoropyrimidines, irinotecan, and oxiplatin, as well as targeted drugs, such as bevacizumab, angiogenesis inhibitors, cetuximab, epidermal growth factor receptor (EGFR) inhibitors, regorafenib, and multikinase inhibitors (7, 9, 10).

In recent years, combinational regimes and targeted drugs have moved rapidly from the bench to the bedside, and many others are currently under investigation in clinical trials to improve the outcomes of patients with CRC, especially those with metastatic disease (5, 11, 12). However, despite these efforts, the 5-year overall survival (OS) rate of patients with CRC ranges from 90 to 10%, highlighting the urgent need for better therapeutic strategies (7, 10, 13, 14).

In the latest few decades, immunotherapy has been proven to aid in the treatment of many solid tumors, notably melanoma and non-small cell lung cancer (15, 16). Immune checkpoint blockade (ICB), such as blocking of programmed death-1 (PD-1), has emerged as a successful approach that provides long-lasting benefits for patients with cancer and significantly improves prognosis (17). Currently, this immunotherapeutic approach is used as the first-line treatment for patients with advanced solid tumors (18–21). However, in CRC, ICB targeting PD-1 or PD-ligand 1 (PD-L1) has shown no clinical benefit. In 2017, pembrolizumab, an anti-PD-1, was approved by the Food and Drug Administration (FDA) as a second-line treatment for patients with mCRC associated with microsatellite instability (MSI-H) (22). In 2020, the treatment paradigm was revolutionized by the FDA approval of pembrolizumab, which was used as the first-line treatment for patients with unresectable or metastatic MSI-H CRC, with no prior systemic treatment for advanced disease; the approval was based on data from the Keynote-177 study (5, 23). However, only a few patients with deficient DNA mismatch repair (dMMR)/MSH-H respond to immunotherapy, and approximately 50% of them rapidly develop primary immune resistance (15–17).

In view of the sustainable clinical responses in the treatment of hematological indications, CAR T-cell therapy against solid tumors has shown poor efficacy and did not achieve the desired outcome (24–28). These unsuccessful outcomes are mainly due to two major obstacles associated with poor response or relapse during CAR T-cell therapy. The first major issue is the lack of suitable target antigens for CAR T-cells in solid tumors; in most individuals, target antigens are present in healthy tissues as well, leading to on-target, off-tumor toxicities (29). The second dilemma is the poor accessibility of target antigens from CAR T-cells, leading to inefficient activation, expansion, and function. Other obstacles are the heterogeneous pattern of tumor antigens expressed by tumor cells, escape mechanisms for evading single tumor antigen-specific CAR T-cells, immunosuppressive tumor microenvironment (TME) (30), CAR T cells non-responsiveness or exhaustion, insufficient infiltration of CAR T-cells into the TME, and metabolic privation. These are implicated in the poor efficacy of CAR T-cell therapy, leading to negative results in clinical trials (31–33).

In this review, we aim to provide state-of-the-art knowledge in preclinical and clinical studies of CAR T-cell therapies for CRC treatment, the most important success-limiting challenges in the application of CAR T-cells and ultimately future directions needed to render this therapeutic tool useful for CRC to achieve efficient clinical translation.

## Biological aspects of CAR T-cell technology

**Figure 1:**
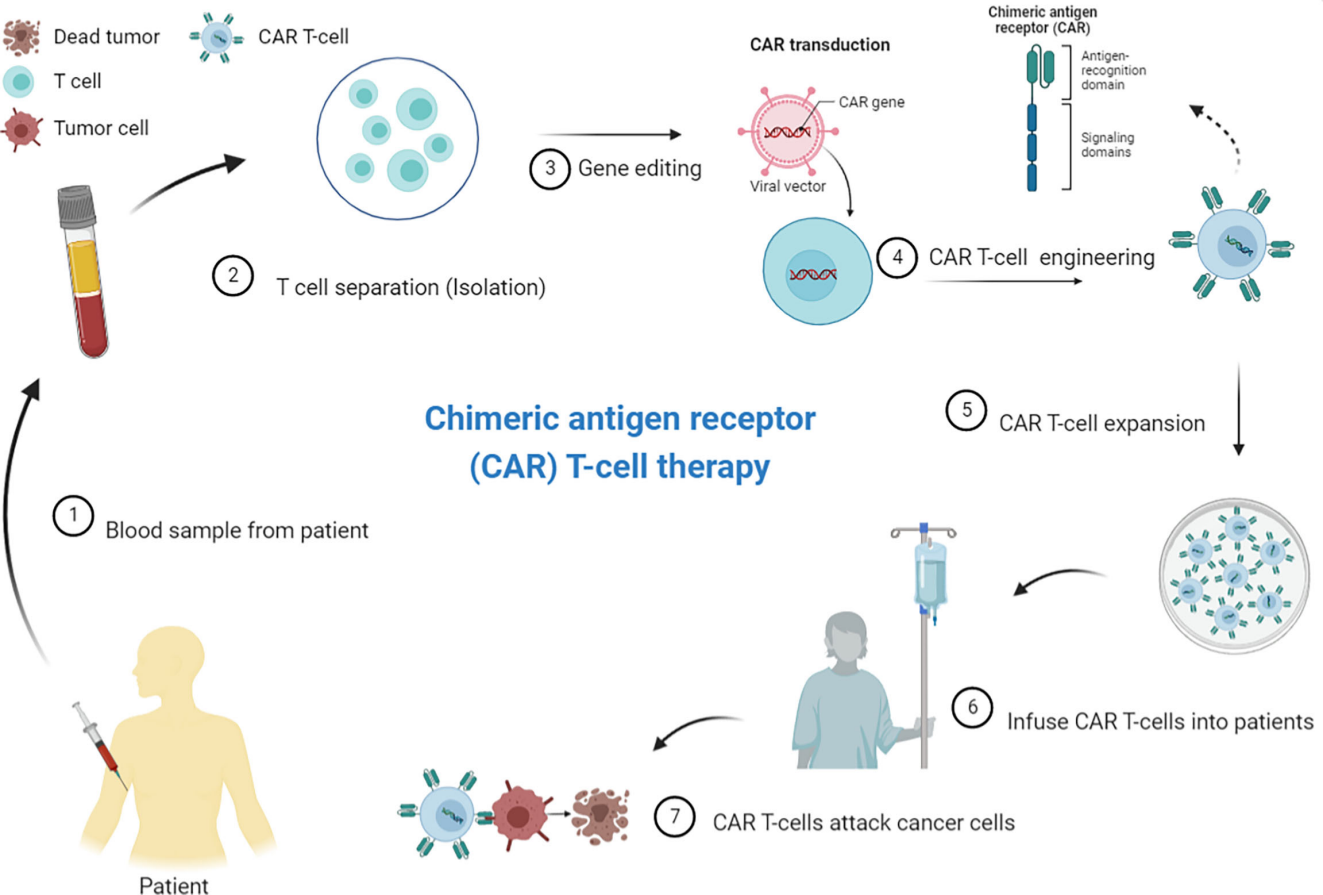
Principles of chimeric antigen receptor (CAR) T-cell therapy/CAR T-cell manufacturing process. CAR T-cell production includes normal T-cell extraction from the patient’s peripheral blood, followed by enrichment and CAR vector delivery, using viral or non-viral vector systems, into the T-cells *in vitro*. The CAR T-cells is subjected to expansion before being re-injected into the patient’s bloodstream. Patients usually receive a lymphodepletion before CAR T-cell administration to improve the expansion of adoptively transferred T-cells. The CAR T-cells proliferate and attack tumor cells that carry the specific antigen against which the CARs are directed (Source: Author’s personal collection, 2021). Created with BioRender.com.

Modified T-cell therapies, including CAR T-cell therapy, have already emerged in the realm of hematologic cancers. CAR T-cell strategy is a personalized immunotherapy based on genetically modified allogeneic or autologous synthetic CAR-expressing T-cells (34). The CAR molecule is composed of extracellular binding moieties, often an antibody-derived single-chain fragment variable or tumor-specific antigen (TSA)-sensing element. Atransmembrane anchor fused to signaling domains from the T-cell-receptor zeta chain complex and costimulatory molecules, such as CD28 and 4-1BB is also found. Specific and direct recognition of tumor antigens through the extracellular domain drives CAR T-cell activation, which results in cancer cell death (35). CARs are most commonly transduced into a patient’s T-cells using lentiviral or gammaretroviral vectors. After the manufacture of CAR T-cells ex vivo, the patient receives lymphodepleting chemotherapy, if needed, followed by CAR T-cell infusion (**Figure 1**).

CAR T-cell receptors are designed to (5) deliver strong activation, proliferation, and survival signals *via* a single binding event (6); recognize tumor antigens in a manner independent of the major histocompatibility complex, bypassing the downregulation of the major histocompatibility complex by certain tumors; and (7) exhibit high affinity even at low antigen density (36–38). The evolution of CAR T-cell strategies from the original design, ectodomain antibody single-chain fragment variable fragment engineered to the T-cell receptor (TCR) ζ-chain, has resulted in several CAR generations. Hence, CAR design has evolved from conventional structures to more sophisticated moieties, enabling combinatorial antigen selection with different signaling abilities (37). Thus, this development supports the production of armored CAR T-cells with substantial enhancement of antitumor efficacy and adequate control of toxicity (39, 40).

The growing demand for CAR T-cells in cancer therapy has been accompanied by an effort to ameliorate a few undesirable aspects of CAR T-cells.

### Control of CAR T-cell hyperactivity and exhaustion by rapidly redirecting intervention and preventing antigen escape

CAR T-cells can amplify or attenuate the immune response due to well-thought out the intracellular domains design, by generating signals of varying strengths, durations, and intensities. An expected complication of this therapy is that excessive CAR T-cell activation drives a cytokine storm, leading to “on-target toxicity.” Another potential issue is “off-target toxicity” to normal cells when the current targets do not allow distinction between normal and cancer cells (37). On-target and off-target toxicities of CAR T-cells need to be monitored and measures taken quickly to abrogate them. In contrast, CAR T-cell exhaustion induced by sustained signaling of chimeric receptors contributes to poor persistence and limits the durability of CAR T-cell immunotherapy.

T-cell exhaustion is a dysfunctional state of differentiated T-cells that differs from anergy and senescence. The exhausted state is a complex phenotype associated with progressive loss of effector functions and poor memory T-cell response (41). This exhausted state is associated with increased expression of multiple inhibitory receptors, including PD1 (42). Another concern about conventional CAR T-cells is fixed antigen specificity, which means that only one TAA can be targeted at a time (37). This limitation harbors the risk of tumor-variant appearance and limits the eficacy of CAR T-cell therapy.

### Identification of specific targeting strategies

CAR T-cells do not follow the classical T-cell–target cell immunological synapse topology. The epitope location, structural dimensions of the target molecule, and extracellular spacer length of CAR allows for the specificity of CAR T-cell dimensional interactions (43, 44). Steric constraints due to the location of the target epitope may require structural modeling of the CAR for adapting/modulating the extracellular spacer length and promoting synapse formation between effector T-cells and tumor cells (45, 46). Other aspects, including the density and accessibility of target molecules to tumor cells, need to be considered (46). In the context of immunological synapse dynamics, tumor cells and their microenvironment underpin specificity and complexity of immune responses through secretion of immunosuppressive soluble factors, such as indoleamine-2,3-dioxygenase-1 (IDO1), PD-L1, and IL-10 (47–50), and recruitment of immunosuppressive cells, such as tumor-associated macrophages and myeloid-derived suppressor cells (51). This immunosuppressive microenvironment limits immune cell homing and infiltration and inhibits effector T-cell activity (52).

The metabolic landscape created by cancer cells in the TME dampens the ability of T-cells to respond (53). The energetic state of the TME and catabolites generated by tumor metabolism play a critical role in T-cell biology, affecting T-cell infiltration, survival, and function (54). The production of immunosuppressive enzymes, such as IDO, tryptophan-2,3-dioxygenase, nitric oxide synthase, and arginase-1, impairs the activation and effector function of T-cells by causing tryptophan and L-arginine deficiency. These aspects of the TME impact CAR T-cell functions and lead to significant clinical implications (55).

According to data from clinical trials on CAR T-cells in solid tumors, immunotherapy outcomes are associated with multiple parameters that reflect cross-talk between the TME and the immune system, including tumor mutational burden (TMB), immunologically hot tumors with inflammatory microenvironments containing high levels of infiltrating cytotoxic T-cells, immune escape mechanisms, timing of administration, and delivery systems of immunotherapeutic agents (56).

**Figure 2:**
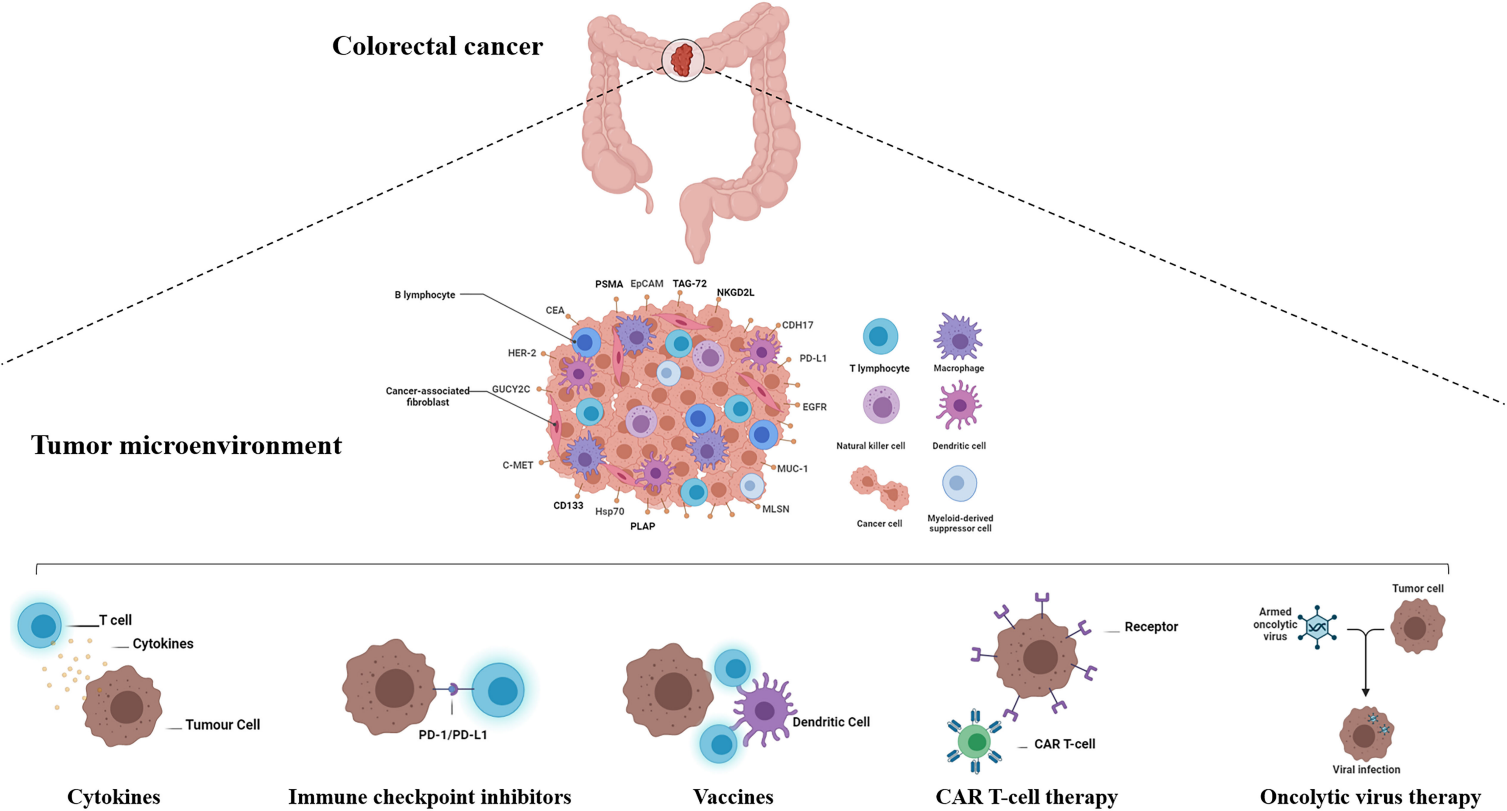
Active and passive immunotherapy strategies in colorectal cancer. The immunotherapy treatments approved in recent years has widened the arsenal against colorectal cancer. Active immunotherapy includes cancer vaccines, checkpoint inhibitors, and oncolytic viruses. Cancer vaccine immunotherapy approaches are based on immunizing patients with cancer against their autologous cancers using either autologous tumor cell-derived vaccines, dendritic cell vaccines, peptide vaccines, DNA vaccines, or viral vector vaccines. Passive immunotherapy includes the use of immune checkpoint blockade, cytokines, and adoptive cell transfer of ex vivo educated immune cells (Source: Author’s personal collection, 2021). Created with BioRender.com.

These considerations indicate that the following strategies are key for the generation of potent CAR T-cells with limited side effects: improving CAR T-cell chimeric receptor flexibility, engineering an appropriate extracellular domain for antigen recognition and binding ability, and improving intracellular signaling capacities. Further understanding of TME variables will help in the design of therapeutic strategies that would enhance the clinical effectiveness of CAR T-cells, especially in solid tumors (**Figure 2**).

## Promising role for CAR T-cells in CRC

CRC is one of the most common malignancies and the fourth leading cause of cancer-related death worldwide (57). According to estimates, the CRC burden is expected to increase by 60% to more than 2.2 million new cases and 1.1 million cancer deaths by 2030 (58). First-line therapeutic approaches for patients with CRC, including surgery and chemotherapy, have long been associated with poor prognosis (59). Even if the response rate to chemotherapy can reach up to 50%, the treatment of far-advanced or recurrent diseases remains modest (60). A better understanding of the pathways contributing to tumor evolution and proliferation has enabled the development of effective and target-selective therapies. Targeted therapies are now being used in daily clinical practice for metastatic CRC with significantly improved survival outcomes (61). Following success with EGFR-targeted therapy (cetuximab) and angiogenesis inhibition (bevacizumab), immunotherapy is now taking center stage and is widely considered one of the most promising potential treatments (61–63). Three CAR T-cell therapies have been approved by the FDA and the European Medicines Agency; all of them target CD19-positive hematological malignancies (axicabtagene ciloleucel, tisagenlecleucel lecleucel, and brexucabtagene autoleucel).

Despite CAR T-cells being promising for hematological malignancies, their therapeutic efficacy in solid tumors, including CRC, remains unproven. Several groups have focused on pre-clinical studies involving CAR T-cell biology, aiming to develop safe therapeutic strategies and validate their use in CRC.

Several CAR T-cell approaches have been developed to target different tumor antigens. TSAs, also called neoantigens, are of high value as therapeutic targets because of their low on-target off-tumor effects. TSAs are dysfunctional peptides derived from the expression of non-synonymous somatic mutations and typically result from tumor-specific mutations; thus, they are only expressed in tumor cells with a high immunogenic potential (64). However, due to the low frequency of TSA mutations among patients with CRC, the investigated CAR T-cells are directed against TAAs, which are expressed in normal cells but overexpressed in tumor cells (64, 65). The different antigens targeted by CAR T-cell therapies in CRC and those reported in preclinical studies are summarized in **Table 1**.

**Table 1:** Antigens targeted by CAR-T cell therapies in CRC and reported in preclinical studies.

IN VITRO STUDIES														
Target		Gen.		Co-St.		Vector		Cell Line	Ratio (Effector : Target)		Result		Ref.	
**CD133**		2nd		4-1BB		LV		SW620	1:1; 5:1		Significant elimination of target cells (% C (5:1): ~40%).		(66, 67)	
**CEA**		2nd		4-1BB		LV		HT29	4:1; 2:1; 1:1; 1:2; 1:4		% C (2:1): ~75%, that significantly increases with rhIL-12 (% C (2:1): ~90%) and releases a higher concentration of IL-2 and IFN- γ.		(68)	
		2nd		CD28		RV		LS174T	1:2:0.02 (MSC)		% C: ~60%, significantly increased in combination with IL7/IL12 expressing MSCs (% C: ~80%).		(69)	
		2nd		CD28		RV		MC38-hCEA-Luc	1:1, 2:1, 5:1		not shown		(70)	
		2nd		CD28		RV		MC-38-CEA-2 (MC-32)	1:2, 1:5, 1:10		CAR-T cells significantly reduce the target cells (% C (1:10): ~90% MC-32), % of apoptosis in CEA CAR with Bcl-xL was decreased compared to CEA CAR alone.		(71)	
**CEA CD30/CEA CD25/CEA**		2nd		CD28		RV		LS174T	3:1; 2:1; 1:2; 1:5		CD30/CEA-CAR-T cells induce higher cytotoxicity (% C (1:2): ~70%) than CEA-CAR-T and CD30-CAR-T. CD25/CEA-CAR-T has similar cytotoxic effects to CEA-CAR-T (% C (1:5): ~15%). Only CD30/CEA CAR-T enhanced the release of perforin and especially granzyme B.		(72)	
**EGFRvIII**		3rd		CD28 4-1BB		LV		DLD-1, HCT116	30:1; 10:1; 3:1; 1:1		% C (10:1): ~80% DLD-1 and 65% HCT116, and increase in caspase 3/7 proteins release. Combination with miR-153 (that inhibits IDO1 expression) enhances CAR-T antitumor activity.		(73)	
**EpCAM**		3rd		CD28 4-1BB		LV		SW480, HT29	2.5:1; 5:1; 10:1		% C (10:1): ~50% SW480 and HT29.↑ Release of IFN- γ and TNF-α.		(74)	
		2nd		4-1BB		LV		SW620 SW480 HCT116 HT29 LoVo	0.5:1, 1:1, 2:1, 4:1, 8:1;16:1		% C (16:1): ~60% SW620, 55% SW480, 50% HCT116, 40% LoVo and HT29. ↑ Release of IFN- γ, IL-2 and IL-6.		(75)	
		3rd		CD28 4-1BB		mRNA		HRT-18G	1:1; 2.5:1; 5:1; 10:1; 20:1		% C (10:1): ~45%. ↑ Release of IFN- γ and granzyme B.		(76)	
		2nd		CD28		LV		HCT116, SW480, A549, RKO	8:1, 4:1, 2:1		EpCAM CAR T cells displayed stronger killing activity against EpCAM-positive cell lines HCT116 and SW480 at E:T ratios of 8:1, 4:1, and 2:1, ↑Release of IFN- γ		(77)	
**GUCY2C**		3rd		CD28 4-1BB		RV		T84	5:1; 10:1		% C (10:1): ~65%. ↑ Release of IFN- γ, TNF-α and IL-2.		(78)	
**HER2**		2nd		4-1BB		LV		HCT116	0.3:1; 3:1; 9:1; 27:1		% C (1:9): ~50%. ↑ Release of IFN- γ, TNF-α, IL-2 and granzyme B.		(79)	
		2nd		4-1BB		LV		DLD-1	1:1, 5:1, 10:1		% C (10:1): ~60%. ↑ Release of IL-2		(80)	
**MSLN**		3rd		CD28 4-1BB		LV		HCT116	2.5:1		Complete elimination of MSLN + tumour cells (~0 of normalised cell index). ↑ Release of IFN- γ and TNF-α.		(81)	
**NKG2DL**		3rd		CD28 4-1BB		Minic. DNA		HCT116, LS174T	5:1; 10:1; 20:1		CAR-T cells significantly reduce the target cells (% C (10:1): ~30% HCT116 and 25% LS174) and also produce significant amounts of IFN- γ and IL-2.		(82)	
**PLAP**		2nd		CD28		LV		LoVo Caco-2 LS123	10:1		High cytotoxic effect and ↑ release of IFN- γ. Combination with α-PD-1, α-PD-L1or α-LAG3 significantly increased C (% C: LoVo cells (CAR-T: ~65%; CAR-T + α-PD-1: 70%; CAR-T + α-LAG3: 80%); LS123 cells (CAR-T: ~65%; CAR-T + α-PD-1: 75%; CAR-T + α-PD-L1: 80%) and IFN- γ release.		(83)	
**TAG-72 CD30/TAG-72**		2nd		CD28		RV		LS-C	1:5; 1:3; 1:2.5; 1:1.2		CD30/TAG-72-CAR-T cells show significantly higher C (% C (1:1.2): ~70%) in comparison with TAG-72-CAR-T cells		(72)	
**Hsp70**		2nd		CD28		RV		LS174T LoVo	1:1, 2.5:1, 5:1, 10:1		CAR-T cells significantly reduce the target cells (% C (2.5:1): ~90% LS174) with significant ratio‐ and time‐dependent increase in GrB and IFN-g release		(84)	
**PD-L1**		2nd		4-1BB		LV		SW480, HCT116	10:1		Combinational therapy (PD-L1-CAR-T cells +CCSC-DC vaccine-sensitized T cells) showed powerful cytotoxicity (%C: (10:1) 33.79% in ALDH^high^ CCSCs SW480) higher than that of the PD-L1-CAR-T cell alone (% C (10:1): 13.88% in ALDH^high^ CCSCs SW480)		(85)	
**IN VIVO STUDIES**														
**Target**	**Gen.**		**Co-st.**		**Mouse model**		**CAR-T Cell Treatment**			**Efficacy**		**Safety**		**Ref.**
**CEA**	2nd		4-1BB		HT29-RFP xenografts (female BALB/c nude mice)		5 × 10^6^ and 1 × 10^7^ cells (IV/2ds) +/rhIL-12			Tumour reduction (day 21) and elimination (day 28) when combined with rhIL-12. ↑ Serum IL-2, IFN- γ and TNF-α.		No obvious body weight loss.		(68)
	2nd		CD28		LS174T xenografts (NSG mice)		2 × 10^6^ cells with 4 × 10^5^ IL7/IL12- expressing MSCs (SC/1d)			Improved tumour suppression and prolonged survival after combined treatment with CAR-T cells and IL7/IL12-expressing MSCs, co-inoculated with the tumour cells.		NR		(69)
	2nd		CD28		LS174 xenografts (Rag2 −/− cG −/mice)		5 × 10^6^ cells (SC/1d)			Significant inhibition of tumour formation after CAR-T cell treatment co-inoculated with the target tumour cells.		NR		(72)
	2nd		CD28		MC38 xenografts (C57BL/6J and B6.SJL male mice)		5.0 ×10^6^ CAR-T (PV or TV/1d)			RD was more effective at controlling tumor growth versus SD. HPRD resulted in increased CAR-T penetration versus LPRD, and suppression of tumor proliferation		NR		(70)
	2nd		CD28		MC32 xenografts (C57BL/6J mice)		3 × 10^6^ CAR-T (IV/1d)			Increased accumulation of CEA-Bcl-xL CAR-T cells in tumor tissues compared to mice injected with CEA CAR-T cells without overexpressing Bcl-xL. Mice receiving CEA-Bcl-xL CAR-T cells had the smaller MC-32 tumor sizes compared to CEA-CAR alone, correlating with enhanced survival		NR		(71)
**EGFRvIII**	3rd		CD28 4-1BB		DLD-1 or miR-153 overexpressing DLD-1 xenografts (NSG mice)		2 × 10^6^ cells (IV/1d)			CAR-T cells eradicated the tumour in 3/5 DLD-1 xenografts and in 5/5 miR-153-overexpressing DLD-1 xenografts.		Little weight change.		(73)
**EpCAM**	3rd		CD28 4-1BB		HT29 or SW480 xenografts (female NOD/SCID BALB/c mice)		2 × 10^7^ cells (SC/1d)			Delay in tumour formation after CAR-T cell treatment co-inoculated with the target tumour cells.		No GvHD and no toxic changes in main organs.		(74)
	3rd		CD28 4-1BB		HRT-18G xenografts (NSG mice)		1 × 10^7^ cells (IP/8ds)			Transient (mRNA) CAR-T cells significantly increase survival of late-stage CRC mouse models.		NR		(76)
	2nd		CD28		HCT116 xenografts (Female NSG mice)		8 × 10^6^ cells (IV/every other day for 4 times)			The combination of EpCAM CAR-T with the Wnt inhibitor-hsBCL9CT -24 suppressed the growth rates of tumors compared with the EpCAM CAR T cells alone, improve infiltration of T cells, downregulate TGF- β and upregulate CXCL10, IFN-c, BLIMP1, and ID2.		No graft-versus-host reactions (diarrhea or rash). No obvious toxic pathologic changes in main visceral tissues (heart, kidneys, liver, lungs, and spleen)		(77)
**GUCY2C**	3rd		CD284 1BB		CT26.hGUCY2C syngeneic mouse model (BALB/c mice) and T84. FLuc xenografts (NSG mice)		1 × 10^7^ murine CD8^+^ CAR-T cells (IP/1d)			Murine CAR-T cells induced tumour reduction in both mouse models.		No intestinal toxicity due to cross-reactions.		(78)
**HER2**	2nd		4-1BB		PDX model (SCID-NPG mice)		2 × 10^6^ cells (IV/1d)			Complete tumour eradication after 2 months and elimination of tumour re-inoculation. ↑ Persistence (16% of CD3^+^ cells are CAR-T cells at day 28).		NR		(79)
	2nd		4-1BB		PDX model (NCG mice)		1 × 10^7^ cells (IV/1d)			HER2 CAR-T cells displayed greater aggressiveness in PDX models, prevents CRC progression, and inhibits distant metastases significantly; persistence (65.8% at day 10)		No obvious pathological changes were found in heart, liver, lung, and kidney sections		(80)
**MSLN**	3rd		CD28 4-1BB		HCT116 xenografts (NCG mice) and PDX model (PDX-col0092 mice)		2.5 × 10^6^ cells (IV/1d)			Xenograft model: tumour reduction and durable response (until the endpoint); persistence (7.5% of CD3^+^ cells are CAR-T cells at day 10). PDX model: reduction in large (1000 mm^3^) and small (50 mm^3^) tumours; MSLN-CAR detected in serum at endpoint.		No significant body weight changes. GvHD: significant hair loss after 120 days in 1/5 PDX mice.		(81)
**NKG2DL**	3rd		CD28 4-1BB		HCT116-Luc xenografts (male NOD/SCID mice)		1 × 10^7^ CD8 ^+^ CAR-T cells (IV/2ds)			Tumour growth suppression and persistence (NKG2D-CAR detected in the tumour sections after 25 days).		Gradual loss of body weight. No toxicity in healthy tissues.		(82)
	1st		–		HCT116-Luc xenografts (NGS mice)		1x10^7^ CAR- γ δ T cells (IP/6ds)			Transient (mRNA) CAR- γ δ T cells delayed tumour growth, but tumours regrowth after treatment.		NR		(86)
**PLAP**	2nd		CD28		LoVo xenografts (male NSG mice)		1 × 10^7^ cells (IV/3ds)			Decrease in tumour growth and persistence (CAR-T cells detected in blood after 16 days).		No decrease body weight and no changes in serum AST, ALT and amylase.		(83)
**PD-L1**	2nd		4-1BB		SW480 xenografts (STOCK-Foxn1nu/Nju mice)		3 × 10^6^ cells (IV/2ds)			Combination therapy ((PD-L1-CAR-T cells +CCSC-DC vaccine-sensitized T cells)) dramatically decreased the tumor burden and volume in mice compared with the control group		NR		(85)
**CDH17**	2nd 3rd		CD28 4-1BB		AOM/DSS-induced primary CRC tumor (C57BL/6)		5 × 10^6^ (IV/2ds)			CDH17CARTs reduce tumor burden and enhance CD3 + T cells infiltration.		No toxicity to normal cells nor autoimmunity		(87)

ALT, alanine aminotransferase: AST, aspartate aminotransferase: C, cytotoxicity: Co-st., co-stimulatory domain: d(s), dose(s): Gen., CAR generation: GvHD, graft-versus-host disease: HPRD, High-pressure regional delivery: IP, intraperitoneal: IV, intravenous: LPRD,Low-pressure regional delivery: LV, lentivirus: Minic., minicircle: MSC, mesenchymal stem cell: NCG, NOD CRISPR Prkdc IL2RG: NSG, NOD/SCID/IL-2R γ c null: NOD, non-obese diabetic: NR, not reported: PDX, patient-derived xenograft: PV, Portal vein: RV, G-retrovirus: Ref., reference; SC, subcutaneous; SCID, severe combined immune deficiency; TV, Tail vein; ~: around.

### Epithelial cell adhesion molecule

One of the first preclinical studies, published by Ang et al., focused on the cytotoxic effects of epithelial cell adhesion molecule (EpCAM or CD326)-directed CAR T-cells. EpCAM is a transmembrane glycoprotein expressed on the surface of normal epithelial cells (88). EpCAM is one of the first cancer stem cell antigens to be reported. Its overexpression is associated with increased cell proliferation, migration, invasion, and tumor metastasis (88, 89). In CRC, EpCAM is overexpressed in more than 90% of all cancer cells and is associated with poor prognosis (89, 90). In the study conducted by Ang et al., EpCAM-targeting CAR T-cells were developed using a humanized monoclonal antibody, and their ability to treat CRC-xenografted mice was assessed (76). This *in vivo* immunodeficient mouse model displayed a phenotype similar to late-stage metastatic cancer in humans, including extensive peritoneal metastasis and ascites production (76). Interestingly, repeated injections of EpCAM-CAR T-cells suppressed peritoneal disease progression in tumor-bearing xenografted mice.

### Carcinoembryonic antigen

Along with EpCAM, carcinoembryonic antigen (CEA) is another studied target of anti-CRC CAR T-cells. CEA is a glycoprotein belonging to the immunoglobulin family and is overexpressed in many types of human cancers, including colon, pancreatic, gastric, lung, and ovarian cancers. CEA is overexpressed in 98.8% of CRC tissue samples and is one of the most important diagnostic and prognostic tumor markers (91, 92). Thus, CEA-targeted therapies hold promise for generating novel therapeutic strategies for CRC treatment. CAR T-cells targeting CEA have demonstrated excellent antitumor activity both *in vitro* and *in vivo*, which was significantly increased when combined with interleukins, such as IL-12 (68). Schlimper et al. generated cytokine-induced killer cells from blood lymphocytes of patients with CRC and transformed them into CEA-targeting cells by engineering a new CAR. Such cytokine-induced killer cells showed enhanced selectivity towards autologous CEA^+^ colon carcinoma cells and increased secretion of proinflammatory cytokines, such as IFN-γ and cytolysis (57, 93). Owing to the potent effects of IL-12 on T-cell functions, Chi et al. designed CEA-specific CAR T-cells and used them in combination with recombinant human IL‐12 to improve CRC tumor responses (68). The authors showed that CAR T-cells used simultaneously with recombinant human IL‐12 significantly enhanced antitumor activity in a xenografted tumor model. These data highlight the importance of adding cytokines to increase CAR T-cell activity in solid tumors.

To improve efficacy against peritoneal metastases, intraperitoneal (IP) delivery of CAR T-cells have been assessed. Compared with systemic intravenous delivery, regional IP infusion of anti-CEA CAR T-cells effectively protects against CEA+ peritoneal tumors and metastasis, enhances the effector memory response and is associated with increased IFNγ production (70). Therefore, it has been suggested that regional delivery improves CAR T-cell penetration deep inside solid tumors without increasing toxicity or overcoming barriers associated with systemic delivery. Furthermore, intratumoral accumulation of myeloid-derived suppressor cells and regulatory T-cells (Tregs) was detected in IP tumors, and the removal of these cells enhanced intra-tumoral T-cell cytotoxicity (94). Combination of IP CAR T-cell delivery and suppressor cell targeting appears to be a viable strategy for circumventing the immunosuppressive microenvironment in solid tumors.

### Epidermal growth factor receptor

In a xenograft model induced by co-inoculation of CAR T-cells and tumor cells, Huang et al. reported complete eradication of the tumor using EGFRvIII-CAR T-cells in combination with miR-153. These results indicate that miR-153 inhibits IDO1 expression in CRC cells and enhances CAR T-cell therapy. Thus, combination of IDO1 inhibitors and CAR T-cells can prospectively serve as an attractive therapeutic strategy for treating CRC and solid tumors (73).

To generate an adaptable gene-based vector that confers immune-cell specificity to various cancer types, Tamada et al. genetically engineered cells to express a CAR that binds a fluorescein isothiocyanate (FITC) molecule (anti-FITC CAR T-cells). The results indicated an improvement in the ability of the CAR T-cells to treat EGFR-positive CRC tumor-bearing mice (95). This versatile model confers T-cell specificity to a tag, which is easily conjugated with various clinically used tumor-reactive antibodies such as cetuximab, rendering the process economical. The results presented by the authors showed that specific interactions between FITC-labeled cetuximab and anti-FITC CAR T-cells in an immunocompromised mouse model, delayed colon cancer growth (57, 95).

### Placental alkaline phosphatase

A pivotal challenge in solid tumor CAR T-cell therapy is the potential toxicity of on-target/off-tumor effects owing to shared antigens in normal tissues. Placental alkaline phosphatase (PLAP) is a TSA that is not expressed in normal tissues but is overexpressed in CRC, rendering it an attractive target for CAR T-cell therapy (96). PLAP has been detected in more than 20% of colorectal adenocarcinomas. To assess the therapeutic potential of PLAP, a second-generation humanized PLAP-CAR T-cell construct was used in a xenograft model of CRC, which showed significantly decreased tumor growth *in vivo* (83). Humanized PLAP-CAR T-cell activity was further amplified by the addition of ICB therapy (anti-PD-1 or LAG-3 antibodies). This suggests that combining CAR T-cell therapy with ICB may be an effective therapeutic approach for CRC. Hence, the synergistic effect of the combination can be achieved through two steps: infiltration of immunogenically silent tumors by CAR T-cells followed by ICB therapy, which reverses CAR T-cell inhibition and restores functional persistence.

### Heat shock protein 70

Similarly, Dezfouli et al. reported that the stress-inducible membrane form of heat shock protein 70 (mHsp70) was an ideal CAR T-cell target for CRC treatment, due to its sufficient affinity, specificity, density, and valence, while being undetectable in normal cells and tissues (97–99). The antitumor activity of anti-Hsp70 CAR T-cells was demonstrated by increased granzyme B degranulation and IFN‐g secretion, associated with enhanced cytotoxicity against CRC cells expressing mHsp70. Furthermore, the greater *in vitro* persistence of anti-Hsp70 CAR T-cells compared to that of normal T-cells promises long-term persistence in a clinical context (84). Despite the promising *in vitro* results, which support the applicability and specificity of the anti-Hsp70 CAR T-cell approach, and the reduced likelihood of off-target effects, further studies are needed for exploring the safety and efficacy of this approach in relevant *in vivo* models.

### Epithelial glycoprotein 40

Daly et al. used genetically modified T-cells to target the CRC-associated antigen EGP40. The CAR construct contained two EGP40-specific monoclonal antibodies fused in line with the Fc receptor gamma-signaling chain (57, 100). Human redirected T-cells specifically recognize and lyse EGP40-expressing colon carcinoma cells (100).

### Guanylyl cyclase C

A major drawback in the development of CAR T-cell therapy is the toxicity to vital organs (78). Guanylyl cyclase C (GUCY2C) is a membrane-bound receptor that is expressed in >95% of CRC metastases (101, 102). The GUCY2C receptor expressed in intestinal epithelial cells triggers signaling pathways that regulate epithelial cell proliferation, differentiation, and apoptosis (103). In a syngeneic murine CRC model with lung metastases, Magee et al. reported that GUCY2C-targeting CAR T-cells provided long-term protection in the absence of intestinal toxicity (104). CAR T-cells exhibited T-cell activation, cytokine production, and enhanced cytolytic activity in an antigen-dependent manner. In a human xenograft model, GUCY2C-targeting CAR T-cells eliminated human CRC metastatic cells, resulting in survival benefits (105).

### Natural killer group 2 member D

Natural killer group 2 member D (NKG2D) is an activating immunoreceptor associated with tumor immunosurveillance owing its ability to recognize tumor cells and initiate an antitumor immune response. In humans, it is expressed on NK cells, invariant natural killer T-cells, CD8+ T-cells, γ δ T-cells, and certain autoreactive or immunosuppressive CD4+ T-cells (106). Ligands for NKG2D are expressed in response to external signals, such as stress or pathogens, and during neoplastic cell transformation; however, they are typically absent in healthy tissues, rendering it a promising CAR candidate (106, 107). Soluble NKG2D ligands impair NKG2D-dependent functions, resulting in an impaired antitumor response. Thus, they are associated with poor clinical prognosis and metastasis (108). High expression of NKG2DL in human colorectal carcinomas and its correlation with improved disease-free survival rationalized the use of NKG2D as a potential immunotherapy target (109). Deng et al. reported the role of third-generation NKG2D CAR T-cells and investigated their cytotoxicity against human CRC cells in a xenograft model (82). In this model, NKG2D CAR T-cells showed significantly high killing activity, inhibited tumor growth, reduced tumor size, and produced longer overall survival with no severe toxicity to vital organs. Moreover, tumor sections from treated mice showed lymphocyte infiltration (82). However, regarding the genetic modification strategy, the authors showed that electroporation of T-cells in the presence of a NKG2D-CAR minicircle DNA vector reduced cell viability and CAR expression in a time-dependent manner (82). This strategy, based on CAR transient expression, produces less toxic effects but requires re-inoculation for achieving stable antitumor activity (76).

### Double cortin-like kinase 1

There is growing evidence that CRC is derived from cancer stem cells, which are slow-cycling, self-renewing, and pluripotent (110–112). This small fraction of the total cell population can undergo epithelial–mesenchymal transition, a key process for metastasis formation and therapeutic drug resistance (113, 114). Double cortin-like kinase 1 (DCLK1), a microtubule-associated protein, maintains stemness in CRC cells, and it is a putative tumor stem cell (TSC) marker in CRC mouse models (115–118). Sureban et al. described DCLK1-targeting CAR T-cells as an efficient therapeutic approach for eradicating CRC stem cells (110). DCLK1-targeting CAR T-cells have been shown to display higher cytotoxicity *in vitro* and exhibit antitumor activity in *in vivo* xenograft mouse models *in vivo*. Intravenous injections of DCLK1-directed CAR T-cells have been shown to reduce tumor size in tumor-bearing mice, strongly suggesting that DCLK1-based CAR T-cells inhibit CRC tumor growth *in vivo* without obvious cytotoxicity to normal tissues (110).

### Mesothelin

The availability of clinically relevant CRC models is essential for CAR T-cell testing and molecular analysis. In this regard, patient-derived xenograft mouse models of CRC have been generated and used in preclinical studies of anti-CRC CAR T-cells. These models reflect the clinical and molecular heterogeneity of patients, and they have been used in two preclinical studies on anti-CRC CAR T-cells (79, 81, 119). One study selected highly expressed mesothelin (MSLN/MESO) as a target for CAR T-cells, and MSLN-CAR T-cells showed significant antitumor efficacy (81). MSLN expressed at low levels in normal tissues acts as a tumor differentiation antigen.MSLN is a glycosylphosphatidylinositol-anchored protein that was first described as a membrane protein expressed in normal and neoplastic mesothelial cells. In subsequent studies, a broader expression pattern has been demonstrated mainly in cancer, with overexpression observed in multiple solid tumor types, including CRC (120). A significant expression of MSLN is significantly expressed in CRC, with up to 60% of cases showing positivity (120–122). In *in vitro* and i*In vivo* models, MSLN overexpression activates the downstream PI3K/AkT, NF-κB, and MAPK/ERK pathways, which promote cancer cell proliferation, migration, and metastasis and hinder apoptosis (120–128). Although the biological significance of MSLN expression in CRC remains poorly understood, it is certain that MSLN plays a positive role in CRC cell proliferation. Furthermore, MSLN has shown potential as a prognostic biomarker and potential target for CRC treatment.

### Human epidermal growth factor receptor-2

In another study, a patient-derived xenograft mouse model implanted with CRC xenograft has been used for assessing the antitumor capacity of human epidermal growth factor receptor 2 (HER2)-CAR T-cells (79). HER2 is an oncoprotein that is overexpressed in a small but relevant proportion of patients with CRC, especially in anti-EGFR-resistant and RAS wild-type tumors (79, 129). HER2-targeted CAR T-cells have been engineered and their *in vivo* efficacy and safety was tested in a patient-derived xenograft model *in vivo* (79, 80). Significant regression of CRC xenografts has been observed, with protection from relapses upon tumor rechallenge in this mouse model. This enhanced efficacy results in a significant survival benefit (79). Consequently, HER2 has become a promising target for CAR T-cell therapy in CRC. Nevertheless, as clinical trials are full of uncertainty, one of the first clinical trials testing third-generation HER2 CAR T-cellsin patients with metastatic cancers (NCT00924287) was terminated after the first patient died of severe acute respiratory failure as a result of the treatment.

### Cadherin 17

Recently, Feng and his coworkers used CDH17 as a target for CAR T-cells. In humans, CDH17 is a CA^2+^-dependent adhesion switch mainly expressed in the intestinal system (130, 131). CDH17 is upregulated in gastrointestinal cancers and is used as a marker for adenocarcinomas of the gastrointestinal system (132, 133). The results demonstrated that CDH17-CAR T-cells eradicated tumors in a xenografted NSG mouse model without damaging healthy normal tissues expressing CDH17 as it is sequestered and hidden between normal cells (87). This study is the first preclinical investigation of CDH17 as a target for CAR T-cell development, and it uncovers a new class of TAAs that are accessible to CAR T-cells in tumors but are masked in normal cells, protecting the latter from CAR T-cell attack. Hence, in solid tumors, re-investigation of previously identified TAAs is of great interest.

As the clinical safety and effectiveness of CAR T-cell therapy are closely associated with the intrinsic quality and fitness of CAR T-cells, comprehensive preclinical investigations of CAR T-cell biological products are crucial. Such investigations are usually performed in different *in vitro* and *in vivo* models that can prove to be powerful and versatile tools for disease modeling and therapeutic experimentation and generate reliable data that indicate the clinical therapeutic potency of CAR T-cell therapies.

Owing to the promising results obtained in preclinical studies, many CAR T-cell therapies developed against CRC are being evaluated for antitumor efficacy and safety in clinical trials.

## CAR T-cell trials in CRC

CAR T-cell therapy has shown great success in hematological diseases, and progress in the clinical evaluation of CAR T-cells has provided evidence for the promising application of CAR T-cells to solid tumors. Currently, CAR T-cell therapy is transitioning from experimental trials to mainstream clinical practice. However, CAR T-cell therapy for solid tumors shows limited efficacy and poor therapeutic response due, in part, to restricted trafficking, antigen escape and/or lack of antigen expression, and inefficient tumor infiltration.

Several CAR T-cell therapies against various CRC antigens have entered clinical trials. In **Table 2**, we summarize the CAR T-cell clinical studies for CRC, reported on the clinicaltrials.gov website.

**Table 2:** Clinical trials of CAR-T cells in patients with colorectal cancer.

Target	Gen.	Co-st.	Use	CRCSubtype	ID	Ph.	n	CAR-T Cell Treatment	Results	Adverse Events	St	Ref.
**CD133**	1st	–	Au.	CRC	NCT02541370	I/II	20	0.5–2 × 10^6^ cells/kg (2 ds)	NA for CRC patients (only reported for HCC patients).	NA for CRC patients (only reported for HCC patients).	C	(134)
**CEA**	2nd	CD28	Au.	CEA+ Liver met.	NCT02416466	I	8	1 × 10^10^ cells/d (3 ds) with IL-2 followed by SIRT	(*n* = 6) 67% PD and 33% SD in the liver, and 17% ND and 83% PD in the extrahepatic.	G 3 (*n* = 6): 33% colitis, 33% fever and 38% reduction in K+. No CRS or neurotoxicity.	C	(135)
	2nd	CD28	Au.	CRC	NCT02349724	I	75	5 DL: 10^5^–10^8^ CAR+ cells/kg (split d: 10%, 30% and 60% per day)	(*n* = 10) 70% SD, 20% PD and 10% NE.	G 2 (n=10): 20% fever (CAR-T related). G 3/4: lymphodepletion related. Minor CRS after high	unk	(92)
	2nd	CD28	Au.	CEA+ Liver met.	NCT02850536	I	5	1 × 10^10^ cells/d (3 ds) with IL-2	(*n* = 1) Complete metabolic response within the liver over 13 months	G 3 (n = 1): dehydration, fever, hyperglycaemia, hypertension, hypokalaemia, hyponatraemia, and hypophosphataemia. No on-target/off tumour SAEs.	C	(136)
	NA	NA	Au.	mCRC	NCT02959151	I/II	20	1.25–4 × 10^7^ CAR+ T cells/cm^3^ tumour bulk (1 d)	NA	NA	unk	NA
	NA	NA	Au.	CRC	NCT03682744	I	18	NA	NA	NA	W	NA
	NA	NA	Au.	CRC	NCT04348643	I/II	40	NA	NA	NA	R	NA
	NA	NA	Au.	Stage III Liver met.	NCT04513431	eI	18	NA	NA	NA	NyR	NA
	NA	NA	NA	Liver met.	NCT05240950	1	18	1×10^6^/kg, 3×10^6^/kg, and 6×10^6^/kg	NA	NA	R	NA
	NA	NA	NA	CRC	NCT05415475	I	36	1-10x10^7^/kg (Intravenous), 1-10x10^7^/kg (intraperitoneal)	NA	NA	R	NA
	NA	NA	NA	CRC	NCT05396300	I	60	3-10x10^6^ cells/kg (Intravenous), 3-10x10^6^ cells/kg (intraperitoneal)	NA	NA	R	NA
**C-Met**	NA	NA	Au.	CRC	NCT03638206	I/II	73	NA	NA	NA	R	NA
**EGFR**	3rd	CD28 4-1BB	Au.	EGFR+ mCRC	NCT03152435	I/II	20	NA	NA	NA	unk	NA
	4th	CD28 4-1BB	Au.	mCRC	NCT03542799	I	20	NA	NA	NA	unk	NA
**EpCAM**	2nd	CD28	Au.	Colon Cancer	NCT03013712	I/II	60	1–10 × 10^6^ CAR+ T cells/kg (1 d)	NA	NA	unk	NA
	NA	NA	Au.	CRC	NCT05028933	I	48	3×10^5^/kg, 1×10^6^/kg, 3×10^6^/kg	NA	NA	R	NA
**HER2**	NA	NA	Au.	CRC	NCT02713984	I/II	0	NA	Reformed CAR structure due to safety considerations	NA	W	NA
	NA	NA	Au.	CRC	NCT03740256	I	45	7 DL: 1–100 × 10^6^ cells (1 d) and oncolytic adenovirus CadVEC	NA	NA	R	NA
**MSLN**	4th	NA	Au.	CRC	NCT04503980	eI	10	4 DL: 1 × 10^5^–3 × 10^6^ cells/kg (1 d)	NA	NA	unK	NA
	NA	NA	Au.	CRC	NCT05089266	1	30	NA	NA	NA	NyR	NA
**MUC1**	NA	NA	Au.	CRC	NCT02617134	I/II	20	NA	NA	NA	unK	NA
	NA	NA	All.	CRC	NCT05239143	I	100	NA	NA	NA	R	(137)
**PSMA**	2nd	CD28	Au.	CRC	NCT04633148	I	35	UniCAR02-T cells with recombinant antibody derivative TMpPSMA	NA	NA	R	(138)
**GCC**	NA	NA	Au.	mCRC	NCT05319314	I	30	NA	NA	NA	R	(139)
**NKG2DL**	NKR-2	End. DAP10	Au.	Liver met.	NCT03310008	I	36	3 DL: 10^8^–10^9^ cells/d (3 ds) and FOLFOX	NA	NA	unK	(140)
	NKR-2	End. DAP10	Au.	Liver met.	NCT03370198	I	1	3 DL: 3 × 10_8_ –3 × 10^9^ cells/d (3 ds)	NA	NA	unK	(141)
	1st	–	All.	Unresec. mCRC	NCT03692429	I	49	3 DL: 1 × 10^8^ –1 × 10^9^ cells/d (3 ds) and FOLFOX	Refractory unresec. mCRC (n = 15): 13% PR, 60% SD and 27% PD.	No treatment-related G ≥ 3 adverse events or GvHD.	R	(142)
	NKR-2	End. DAP10	Au.	CRC	NCT03018405	I	146	3 DL: 1–3 × 10^9^ cells/d (3 ds)	NA	No dose-limiting toxicity	unk	(143)
	NA	–	All.	CRC	NCT04107142	I	10	3DL:3 ×10^8^–3 ×10^9^ CAR-γδ T cells/d (4 ds)	NA	NA	unk	NA
	2nd	4-1BB	Au.	Colon Cancer	NCT04270461	I	0	1–10×10^6^ cells/kg	Study withdrawn because of administrative reasons.	NA	W	NA
	2nd	4-1BB	Au.	CRC	NCT04550663	I	10	NA	NA	NA	NyR	(144)
	NA	NA	NA	Liver met.	NCT05248048	eI	9	NA	NA	NA	R	NA
	2nd	4-1BB	Au.	CRC	NCT05382377	eI	18	NA	NA	NA	R	NA
	1st	End. DAP10	All.	Unresec. mCRC	NCT04991948	I	34	NA	NA	NA	R	NA

ANR, active not recruiting: Au., autologous: All., allogeneic: C, completed: CRC, colorectal cancer: CRS, cytokine release syndrome: d(s), dose(s): DL, dose levels: eI, early phase I: End., endogenous: G, grade of toxicities: Gen., CAR generation: GvHD, graft-versus-host disease: HCC, hepatocellular carcinoma: mCRC, metastatic CRC: M, median: n, patient number: met., metastasis: NA, not available: NE, not evaluable: ND, not detectable: NyR, not yet recruiting: Ph., phase: PD, progressive disease: PR, partial response: R, recruiting: Ref., reference: SD, stable disease: SIRT, selective internal radiation therapy: TIM, T-cell receptor inhibitory molecule: unk, unknown: Unresec., unresectable: W, withdrawn.

In their first attempts to apply CAR T-cell therapy to CRC, several groups have conducted clinical trials to assess the anticancer activity of CAR T-cells directed against HER2, tumor-associated glycoprotein 72 (TAG-72), CEA, or CEACAM5 (33, 92, 145, 146). One of the first clinical trials to evaluate CAR T-cell therapies for CRC targeted HER2 in a patient with colon cancer that was metastatic to the lungs and liver and refractory to multiple standard treatments. HER2-specific CAR T-cells were developed on the basis of the widely used humanized monoclonal antibody trastuzumab. Unfortunately, the patient who received the HER2-specific CAR T-cell infusion experienced respiratory distress with a cytokine storm and died of CAR T-cell-related toxicity (33).

No significant therapeutic benefits were observed with CAR T-cells, which were directed against the other three targets (TAG-72, CEA, or CEACAM5) (92, 145, 146). In another study, Hege et al. reported the results of one of the first CAR T-cell trials for metastatic CRC. The study was based on TAG-72-targeting CART72 cells (145). Two phase 1 trials were conducted using systemic (C9701) or regional hepatic artery infusion (C9702) CART72 delivery routes. Despite its safety profile, CART72 exhibited limited persistence in the blood because of an anti-CAR immune response and incomplete penetration into the tumor masses (145).

A phase I trial was conducted in CEA-positive patients with CRC presenting metastases for evaluating the safety and efficacy of CEA-targeting CAR T-cells (92). The endpoint showed good tolerability of CEA CAR T-cells even at high doses, persistence of CAR T-cells in peripheral blood, and eficacy in most treated patients (92). No significant beneficial clinical response was observed upon CEACAM5-specific CAR T-cell use with dose escalation in a phase I study (146). Respiratory toxicity combined with the lack of demonstrable clinical efficacy resulted in the premature closure of the trial.

Several trials are being conducted for assessing the safety and efficacy of CAR T-cells in CRC treatment. More than 50% of ongoing CAR T-cell clinical trials are in East Asia. A phase I, open-label clinical trial is underway for testing the safety and efficacy of EGFR CAR T-cells in the treatment of metastatic CRC (147, 148). To enhance EGFR CAR T-cell cytotoxicity, persistence, and to overcome a hostile TME in CRC, fourth-generation EGFR-IL12 CAR T-cells, also known as T-cells redirected for universal cytokines (TRUCKs), are engineered to secrete IL-12, a proinflammatory cytokine EGFR-IL12 TRUCKs express IL-12 upon activation and deliver it to enhance local antitumor immune responses, such as activating NK cells and repolarizing macrophages. The maximum tolerated doses and safety of EGFR-IL12 CAR T-cells are being currently assessed in patients with metastatic CRC (147, 148).

The other fourth-generation CAR T-cell therapy tested in CRC is αPD-1-MESO CAR T-cells, which exhibit inducible production and secretion of anti-PD-1 nanobodies (NCT04503980). A Chinese trial is underway to test an αPD-1-MESO CAR T-cell therapy in MSLN-positive advanced solid tumors (colorectal and ovarian cancer). During CAR T-cell production, patients receive a premedication regimen (cyclophosphamide) to deplete lymphocytes. The estimated completion date of the study is June 2022.

Ongoing trials are investigating the use of CEA-specific CAR T-cells in CEA-positive CRC. The objective is to confirm efficacy and safety and to set the appropriate doses and infusion plan (149–153). Another goal of these studies is to establish adverse events, most notably the cytokine release syndrome. Administration protocols include systemic and hepatic transarterial administration, vascular intervention, or intraperitoneal infusion, and the results are awaited (149–153). A new combinatorial strategy using HER2-specific CAR T-cells and an oncolytic adenovirus (CAdVEC) is under clinical investigation (154). Oncolytic adenoviruses replicate and spread specifically inside tumors, enhancing their cytotoxicity, improving tumor penetration, and reversing immune suppression. CAdVEC is a modified oncolytic adenovirus that contains various immunostimulatory molecules (155). Phase 1 trials are currently investigating the efficiency and safety of combining HER2 CAR T-cells with oncolysis (154). The intratumoral administration of CAdVEC is thought to promote an anti-tumorigenic proinflammatory TME that triggers recruitment and local expansion of the infused HER2 CAR T-cells. A phase I study of MUC1+ relapsed or refractory solid tumors was conducted for determining the safety and efficacy of MUC1 CAR T-cell therapy (156). In the phase I/II multi-target gene-modified CAR T-cell/TCR T-cell trial, c-MET was chosen as a target for CRC. Overexpression of c-MET in patients with CRC predicts poor survival (157). The safety and efficacy of CAR T-cells directed against EpCAM and CD133 are being explored in two clinical trials (158, 159). Preliminary data with CD133 CAR T-cells are encouraging, and they further indicate that CD133 can be an interesting target for relapsed or refractory CRC. NKG2D receptors bind to stress-induced ligands that are upregulated in infected and transformed, stressed cells. NKR-2, a second-generation autologous CAR T-cell line, uses the NKG2D complex to deliver primary and costimulatory signals. In contrast to classical target-specific CAR T-cells, NKR-2 recognizes various ligands on tumor cells and exhibits the potential to target several tumors (160). In addition to direct recognition of tumor cells, NKR2 is expected to target NKG2D-positive non-tumor cells within the stroma, tumor blood vessels, and immunosuppressive TME, which disrupts the support mechanisms essential for tumor cell survival and growth (161). Two clinical trials are currently assessing the safety and efficiency of NKR-2 CAR T-cells in patients with CRC, and the results are eagerly awaited. The first trial investigated the hepatic transarterial administration of NKR-2 CAR T-cells in patients with CRC presenting unresectable liver metastases, while the second investigated the safety and efficiency of NKR-2 cells combined with chemotherapy in resectable CRC liver metastases (162, 163).

CYAD-101 is an allogeneic CAR T-cell therapy engineered to co-express NKG2D-based CAR T-cells and the novel inhibitory peptide TCR inhibitory molecule (TIM). The expression of TIM prevents graft-versus-host disease by reducing the TCR signaling complex (164). In an open-label phase I trial, CYAD-101 treatment was administered simultaneously with FOLFOX chemotherapy in patients with unresectable metastatic CRC for assessing its safety and tolerability, clinical activity, and cell kinetics (165).

To date, although no particularly encouraging results have been reported for CAR T-cell therapy in CRC, several promising preclinical studies have shown success. Several major hurdles must be overcome to achieve the expected effect and eficacy in clinical trials. In the application of CAR T-cell therapies for CRC, the physical barrier and immunosuppressive microenvironment are the main hurdles in the achievement of desirable efficacy. Elegant strategies are currently under development for improving CAR T-cell function in solid tumors by prolonging their persistence, trafficking, tumor inflltration, and tumor elimination. For precise tumor targeting, potential solutions can be used, such as targeting T-cells with tandem CARs, universal CARs, affinity tuning, and regulation of CAR expression levels (166). In addition, preliminary data have provided promising evidence for combination therapies of CAR T-cells and other immunotherapies, chemotherapy, or radiotherapy (167).

## Discussion

### Lessons for the future

The efforts of CAR T-cell research teams have yielded outstanding results for the treatment of leukemia (168–174). However, clinical evaluation of this new immunological weapon for fighting solid cancers is surrounded by disappointment. Past experiences, notably with ICIs, have highlighted significant contribution of multiple factors that significantly contribute to the strengths and weaknesses of immunotherapies and forecast their clinical outcome in solid cancer. These aspects must be considered when identifying the therapeutic strategies for solid tumor treatment.

Nevertheless, it must be considered that even for hematological malignancies with complete remission rates as high as 90%, patients remain at risk of relapse because of the poor persistence of CAR T-cells *in vivo*. Limited CAR T-cell persistence is closely related to the immunological properties of the cellular infusion product.

In the last decade, cancer treatment has undergone a major revolution owing to therapies that target the TME. Deep characterization of the immune response and TME, a continuously evolving entity, along with the molecular and phenotypic analysis of tumor cells, is an attractive strategy for cancer treatment. Using accumulated data from recent immunotherapy studies, notably ICI, to design more effective CAR T cells, can be of a great interest.

### Acting on the TME: hot, cold, and in-between tumors

The complex spatiotemporal changes in the TME complicate cancer treatment by driving various responses to immunotherapies. The landscape of tumor-infiltrating immune cells defines prognosis and treatment efficacy (175, 176), supporting the idea that treatment strategy designs need to be guided by mapping the composition and functional state of immune cell infiltrates (177). Schematically, the TME can be broadly classified into cold (non-T-cell inflamed) or hot (T-cell inflamed), on the basis of accumulation of proinflammatory cytokines and T-cell infiltration (178). Commonly, hot tumors, characterized by T-cell infiltration and immune activation signatures, exhibit better response to ICB treatment (179). Meanwhile, cold tumors lack such infiltrates because of several factors: poor tumor antigenicity, impaired antigen presentation, T-cell activation failure, and/or lack of T-cell homing into the tumor bed. A limited number of T-cells surround cold tumors and localize to the tumor periphery with poor T-cell activation and infiltration (180, 181). Thus, the high-inflammatory state of the TME is associated with high TMB and increased tumor-related antigenicity, resulting in T-cell tumor-infiltration, amplification, and tumor necrosis induction (178, 182). Remarkably, CRC tumors with MSI and a higher mutational burden respond well to ICI-based immunotherapy (183–186). Primary lesions, such as metastatic colon cancer, are highly enriched in immune infiltrating cells with increased mutational load, and a high immunoscore is associated with a significantly lower number of metastases (187).

Several research teams are focused on converting cold tumors into hot tumors to render them more receptive to immunotherapies, leading to an objective antitumor response. The second goal was to prevent hot tumors from cooling. In this way, creating a beneficial TME goes through vascular bed remodeling to enhance the circulation of CAR T-cells and activate the immune response at the tumor side. Innate immune sensing and signaling, dysregulated oncologic pathways, altered cellular metabolism, and epigenetic changes in the TME must be integrated as tightly as possible into the global antitumor strategy. Immunogenic cell death induced by chemo-, radio-, and targeted therapies plays a key role in stimulating altered antitumor immune responses, potentially converting non-inflamed cold tumors into hot tumors (188–192). Hence, the future of antitumor combination therapies lies in well-designed sequential strategies that successfully prepare patients for immunotherapeutic regimens, including CAR T-cell adoptive cell transfer (193, 194). Tumor immune microenvironment characterization can help identify good responders to immunotherapies, such as CAR T-cell therapy.

### Implementing TMB quantification for predicting CAR T-cell therapy response

Currently, 28 ongoing clinical trials are evaluating TMB as a stratification biomarker for predicting immunotherapy responses (195–197). The frequency of tumor mutations varies widely across, and within cancer subtypes (198). Greater mutational load, notably nonsynonymous mutations, increases the possibility of neoantigen generation and expression by tumor cells, allowing the conversion of immune tolerance to antitumor immune responses. Consistent with this hypothesis, TMB has been shown to predict the clinical benefit of anti-PD-1/PD-L1 immunotherapy (199, 200). Patients in the immune desert cluster with a low T-cell infiltration microenvironment respond less favorably to ICI therapies (201–203). Moreover, several recent preclinical and clinical reports have clearly highlighted the major role of neoantigen-specific T-cell activity in recognizing and eliminating cancer cells (204–213). Several studies have reported a highly significant association between neoantigen burden and clinical benefits (183, 214–216). Therefore, TMB has emerged as a powerful determinant in response to immunotherapies.

Based on current data and trends, TMB appears to be a robust predictive biomarker of the response to immune checkpoint immunotherapy. Generally, two types of cancer tend to harbor high TMB: (i) tumors induced by carcinogenic and mutagenic substances and (ii) tumors caused by germline mutations in DNA repair and replication factors. Thus, TMB promises to be useful for MSI-H CRCs, marked by frequent mutations in dMMR and DNA replication genes (195, 196). The model combining TMB with other immunotherapy biomarkers provides a promising method with greater predictive power to identify immunotherapy responders and predict outcomes.

In dMMR and MSI-H CRC tumors, neoplastic foci present lymphoid tertiary structures deeply infiltrated with activated CD8^+^ T-cells (217–219). Consistent with this observation, dMMR cancers are sensitive to checkpoint inhibitors, leading to pembrolizumab approval for MSI-H or dMMR cancers (22, 220, 221). In colon cancer, the potent local immune response associated with high mutational load allows neoantigens to be targeted by T and B lymphocytes with an appreciable clinical antitumor reactivity under ICI treatment (183, 222–224).

### Future directions for application of CAR T-cells in CRC treatment

The complex TME plays an essential role in the clinical response of solid tumors to CAR T-cell therapy. Thus, the inhibition of proliferation and/or lack of persistence can limit effective antitumor immune response and lead to the poor treatment response in CRC. The immunosuppressive microenvironment plays a decisive role in inhibiting CAR T-cell persistence in the tumor milieu. On the one hand, immunosuppressive cells and cytokines hamper the function of CAR T-cells by blocking them from tumor sites or inducing apoptosis (36). On the other hand, the immunosuppressive microenvironment lacking the cytokines necessary for T-cell persistence can foster the apoptotic pathway.

Once at the tumor site, CAR T-cells can undergo activation-induced cell death upon functional activation and proliferation/expansion. B-cell lymphoma-extra-large (Bcl-xL), a critical regulator for T-cell survival, has been reported to contribute to CAR T-cell persistence and antitumor activity (225–227). Interestingly, the inclusion of exogenous Bcl-xL gene into a second-generation anti-CEA CAR retroviral construct promoted CAR T-cell persistence both *in vitro* and *in vivo*, with enhanced antitumor potency and sustained survival in a CRC mouse model. The novel CAR T-cell platform which is based on the exogenous expression of persistent genes, can help overcome the lack of persistence of CAR T-cells in solid tumors.

Exploring better combination therapies is another approach to improve CAR T-cell efficacy and decrease their shortcomings for solid-tumor treatment. Emerging novel combined CAR T-cell therapies and immunotherapy or small-molecule drugs have shown promise in preclinical studies, and they can overcome the limitations of CAR T-cells targeting solid tumors. The combination of EpCAM CAR T-cells with the Wnt pathway inhibitor hsBCL9CT-24 has shown a synergistic effect against EpCAM-positive colon cells *in vitro* and *in vivo*. The combined strategies modulate the TME to induce the inflltration of T-cells, effector function and prevent the exhaustion of CAR T-cells (77). Induction of antibody-dependent cell cytotoxicity (ADCC) *via* the Fc-gamma receptor is another multi-target CAR T-cell approach that enables ADCC to CAR T-cells. Hence, by endowing CAR T-cells with ADCC activity *via* the Fc-gamma receptor IIIa (CD16), they exhibit sustained proliferation and cytotoxicity to antibody-targeted cancer cells (228). In CRC, CD16-CAR T-cells combined with cetuximab decreased the viability of KRAS-mutated HCT116 CRC cells *in vitro*, decreased tumor growth in a SCID mouse model, and increased disease-free survival (229). Recently, dendritic cell (DC) vaccines have been studied in combination with immunotherapy strategies, such as those targeting PD-1/PD-L1. Regarding cancer stem cell resistance to conventional therapies, the synergistic killing effects of PD-L1-CAR T-cells and CRC stem cell (CCSC)-dendritic cell vaccine-sensitized T-cells in CCSC have been investigated. A study has shown that PD-L1 is highly expressed in CCSCs and that PD-L1-CAR T-cells specifically recognize the PD-L1 molecule on CCSCs (85). In this study, combined therapy markedly relieved the tumor burden in mice compared to monotherapy with PD-L1-CAR T-cells or CCSC-DC vaccine. This showed that there was moderate tumor remission both *in vitro* and *in vivo*.

Finally, combination therapies are being developed for improving immune response, minimizing on-target off-tumor toxicities, and transforming immunologically “cold” to “hot” tumors (230). A combination of CAR NK and CAR T-cells can harness the advantages of both CAR NK and CAR T-cells, allowing for both rapid and persistent killing while potentially minimizing the toxicities associated with CAR-T cells (228, 231–233). The development of these therapies is poised to be the next frontier in immunotherapy as it will result in more robust immune attacks and effective clinical outcomes.

## Conclusions

Data continues to accumulate supporting CAR T-cells as a promising immunological form of cancer immunotherapy. In hematological malignancies, this concept has considerably improved patient care. However, CAR T-cell therapy also has substantial limitations, including the requirement of certified laboratories and clinical centers with specifically trained personnel to manage the side effects. The use of CAR T-cells in solid tumors faces additional challenges, including inefficient T-cell trafficking and a hostile TME. Overcoming these limitations would enable the achievement of the full potential benefit of this novel approach.

CRC is one of the few cancers in which immunotherapy has shown limited promise. CAR T-cell therapies could be very beneficial in CRC, as suggested by preclinical and early phase clinical studies. However, several questions remain unanswered (1): What is the appropriate target for CAR T-cells? (2) what is the right therapeutic strategy with regard to novel checkpoint inhibitors and monoclonal antibodies: a combination of immunotherapies (ICB, virotherapy, IDO inhibitor, or CAR T-cells) or immunotherapies administered in sequence? The efficacy and safety of cancer treatment appear to depend on the drug combination, timing, and sequence of administration. The treatment schedule design should be driven by changes in the tumor-immune microenvironment. Ongoing clinical trials can answer these questions and provide insights into the spectrum of patients who can benefit from this treatment.

## Author contributions

BG and AEG conceived and drafted the manuscript. AG designed the figures. BG and SK designed the tables. AB made substantial contributions to manuscript modifications. All authors contributed to the article and approved the submitted version.

## Funding

This work was supported by the Moroccan Ministry of Higher Education, Research and innovation through a “PPR1” project and by the Moroccan Ministry of Higher Education, Research and innovation and the Digital Development Agency "ADD” through an “Al-khawarizmi” project.

## Acknowledgments

We would like to thank Konala Priyanka Reddy at London North West Healthcare NHS Trust Harrow on the Hill, United Kingdom who helped us to improve the linguistic quality of the manuscript.

## Conflict of interest

The authors declare that the research was conducted in the absence of any commercial or financial relationships that could be construed as a potential conflict of interest.

## Publisher’s note

All claims expressed in this article are solely those of the authors and do not necessarily represent those of their affiliated organizations, or those of the publisher, the editors and the reviewers. Any product that may be evaluated in this article, or claim that may be made by its manufacturer, is not guaranteed or endorsed by the publisher.

## Glossary

**Table:**

ADCC	Antibody Dependent Cell Cytotoxicity
Bcl-xL	B-cell lymphoma-extra-Large
CAR	Chimeric Antigen Receptor
CDH17	Cadherin 17
CEA	Carcinoembryonic Antigen
CRC	Colorectal Cancer
CCSCs	Colorectal Cancer Stem Cells
DCLK1	Double Cortin-Like Kinase 1
EGFR	Epidermal Growth Factor Receptor
EGP40	Epithelial Glycoprotein 40
dMMR	Deficient Mismatch Repair
EGFR	Epidermal Growth Factor Receptor
EpCAM	Epithelial Cell Adhesion Molecule
FDA	Food and Drug Administration
FITC	Fluorescein IsoThioCyanate
GUCY2C	Guanylyl Cyclase C
HER2	Human Epidermal Growth Factor Receptor 2
Hsp70	Heat shock protein 70
ICB	Immune Checkpoint Blockade
ICIs	Immune Checkpoint Inhibitors
IDO	indoleamine-2, 3-dioxygenase
IP	Intra-Peritoneal
MSI	Microsatellite Instability
MSLN	Mesothelin
NKG2D	Natural Killer Group 2 member D
NOS	Nitric Oxide Synthase
PD1	Programmed Death-1
PLAP	Placental Alkaline Phosphatase
TAA	Tumor Associated Antigen
TAG-72	Tumor-Associated Glycoprotein 72
TCR	T-cell Receptor
TIM	T-cell Receptor Inhibitory Molecule
TMB	Tumor Mutational Burden
TME	Tumor Microenvironment
Treg	Regulatory T-cells
TRUCKs	T-cells Redirected for Universal Cytokine
TSA	Tumor-specific Antigen
TSC	Tumor Stem Cell
